# Remote estimation of overwintering home ranges in an elusive, migratory nocturnal bird

**DOI:** 10.1002/ece3.5723

**Published:** 2019-10-22

**Authors:** Christopher M. Tonra, James R. Wright, Stephen N. Matthews

**Affiliations:** ^1^ School of Environment and Natural Resources The Ohio State University Columbus OH USA

**Keywords:** *Antrostomus vociferous*, eastern whip‐poor‐will, full‐annual cycle, land cover, nightjar, winter

## Abstract

Due to a long running research bias toward the breeding season, there are major gaps in knowledge on the basic nonbreeding ecology of many species, preventing a full‐annual cycle focus in ecology and conservation. Exacerbating this problem is the fact that many species are extremely difficult to detect outside of breeding. Here, we demonstrate a partial solution to this problem by using archival GPS tags to examine the overwintering ecology of a migratory nocturnal bird, the eastern whip‐poor‐will (*Antrostomus vociferous*). We deployed tags on 21 individuals and were able to recover 11 (52%) one year later. Tags collected high precision (approx. 10 m) points throughout the nonbreeding period. With continuous time movement models, we used these data to estimate overwintering home ranges. All individuals exhibited at least one bounded home range during this phase of the annual cycle, three of eleven had two wintering locations, and home range area ranged from 0.50 to 10.85 ha. All overwintering home ranges contained closed‐canopy forest land cover (42%–100%), and no other land cover type represented >40% of any home range. We found some evidence, with caveats, that total edge within the landscape surrounding the home range was negatively related to home range area. The prevalence of contiguous closed‐canopy forest cover in overwintering home ranges contrasts with apparent breeding habitat preferences, which includes clear‐cuts and other, more open, habitats. This study is the first to reveal key aspects of overwintering space use in this species by using archival GPS to overcome both logistical and methodological limitations. Expanded use of such technology is critical to gathering basic ecological and distributional data, necessary for achieving a more complete understanding of full‐annual cycles of animal populations.

## Graphical abstract

By using archival GPS, for the first time we have been able to map home ranges and quantify land cover for a nightjar (Family Caprimulgidae) during the nonbreeding season. We found contrasting patterns of habitat use with the breeding season and high variability in home range area. This is critical natural history information for understanding the full‐annual cycle of this guild.

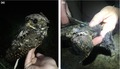

## 1 INTRODUCTION

Research bias toward the breeding season has been a chronic problem in animal ecology, hindering a more complete understanding of where and when populations are limited (Marra, Cohen, Loss, Rutter, & Tonra, 2015). There are a multitude of reasons this bias has perpetuated for several decades, including a lack of emphasis on the importance of nonbreeding stages (e.g., migration, stationary nonbreeding) to population dynamics (e.g., Marra, Studds, et al., 2015) and sublethal impacts of these seasons on breeding (e.g., carryover effects; Harrison, Blount, Inger, Norris, & Bearhop, 2011). However, research on nonbreeding periods is often limited due to the logistical challenges of studying individuals at a time when they are most elusive and, for migratory animals, redistributing their populations (Webster, Marra, Haig, Bensch, & Holmes, 2002). Therefore, in order to improve our understanding of full‐annual cycle ecology, it is paramount that researchers apply emerging research technology and techniques to overcome these barriers.

One of the key elements to understanding population limitation and the basic ecology of animals is quantifying variation in space use, which can influence reproduction and survival (Marzluff, Millspaugh, Hurvitz, & Handcock, 2004). Home ranges can vary in size and position depending on intrinsic factors of individuals, such as body size (Jetz, 2004), as well as environmental factors, such as food availability (Potts, Bastille‐Rousseau, Murray, Schaefer, & Lewis, 2014). Determining the drivers of home range characteristics is necessary for determining the impact of habitat characteristics on carrying capacity through density (e.g., Marra, Studds, et al., 2015). Thus, understanding how home range characteristics vary across the full‐annual cycle can reveal differential space‐use needs (e.g., Webb, Marzluff, & Hepinstall‐Cymerman, 2012) and provide critical information about resource use within changing habitat contexts. However, basic information on the distributions of animals throughout the annual cycle often precludes examination of basic ecology (Marra, Cohen, et al., 2015).

Lack of geographic and distributional information is especially problematic for migratory species. Migratory populations redistribute themselves throughout their annual cycle and can exhibit varying levels of migratory connectivity, from weak (i.e., mixing extensively) to strong (i.e., remaining discrete; Cohen et al., 2018; Webster et al., 2002). Nonbreeding distributions, and therefore habitats, may vary among breeding populations, potentially explaining regional variation in population trends (e.g., Kramer et al., 2018). Thus, when determining drivers of population dynamics across the annual cycle, it is critical that space use of animals from the population of interest is measured. While tracking animals in this way has long been possible in larger animals, using technologies such as ARGOS satellite tags (e.g., Battley et al., 2012), tracking small animals (<50 g) has largely been limited to the use of archival light‐level geolocators (Stutchbury et al., 2009). However, location uncertainty in the hundreds of kilometers (Fudickar, Wikelski, & Partecke, 2012) precludes the use of light‐level geolocators in estimating home ranges.

Although in many cases information on space use is available to inform habitat requirements for populations during breeding, often it is lacking for the nonbreeding period, when species are more difficult to locate/observe. This can be especially true for crepuscular and nocturnal animals, as most detection methods rely on vocalizations (e.g., Zwart, Baker, McGowan, & Whittingham, 2014). For some species, vocalizations occur independent of annual cycle stage, facilitating year‐round detection (e.g., echolocation in bats; O'Farrell, Miller, & Gannon, 1999). However, in birds, most nocturnal species do not vocalize outside of the breeding season (e.g., eastern whip‐poor‐will *Antrostomus vociferus*, Ridgely & Gwynne, 1989; northern saw‐whet Owl *Aegolius acadicus*, Rasmussen, Sealy, & Cannings, 2008) or there is no information on nonbreeding vocalizations (e.g., common nighthawk *Chordeiles minor*, Brigham, Ng, Poulin, & Grindal, 2011). Thus, due to their difficulty to locate and capture, using technologies such as VHF transmitters to quantify space use, which require in situ work on the nonbreeding grounds, is often nearly impossible to implement, as basic distributional data of most populations are lacking (e.g. Holyoak, 2001).

Collectively, these challenges have severely limited our understanding of migratory nocturnal animals' nonbreeding ecology, and in turn, our ability to effectively develop conservation strategies. Fortunately, the recent development of archival global positioning system (GPS) tags offers a potential solution for many species (e.g., Hallworth & Marra, 2015; Siegel, Taylor, Saracco, Helton, & Stock, 2016). These tags weigh as little as 1 g and can collect up to 70 points using GPS satellites with approximately 10 m location uncertainty. Thus, these tags can be placed on individuals from a population of interest and, if recovered, provide high‐resolution location data from the nonbreeding grounds. These data can then be used to determine population‐specific space‐use strategies (e.g., maintenance of a bounded home range), as well as habitat characteristics (e.g., land cover within a home range). Using recently developed space‐use models that incorporate movement rate and distance between successive points, this number of precise locations should be sufficient to estimate home ranges, regardless of autocorrelation among locations (e.g., continuous time movement models; Calabrese, Fleming, & Gurarie, 2016).

One taxonomic group that would greatly benefit from this approach is the diverse group of birds known as nightjars (Family Caprimulgidae). Nightjars are crepuscular/nocturnal insectivores, primarily migratory when found in the temperate zone, and are chronically understudied during the nonbreeding period when they are difficult to detect (Holyoak, 2001). In addition, nightjars belong to the fastest declining foraging guild of birds in North America, aerial insectivores (Michel, Smith, Clark, Morrissey, & Hobson, 2016; Spiller & Dettmers, 2019). Given these declines and the difficulty in quantifying their habitat use throughout the annual cycle, it is critical that methodological approaches be found that can address conservation issues surrounding both nightjars and similar animals.

**Figure 1:**
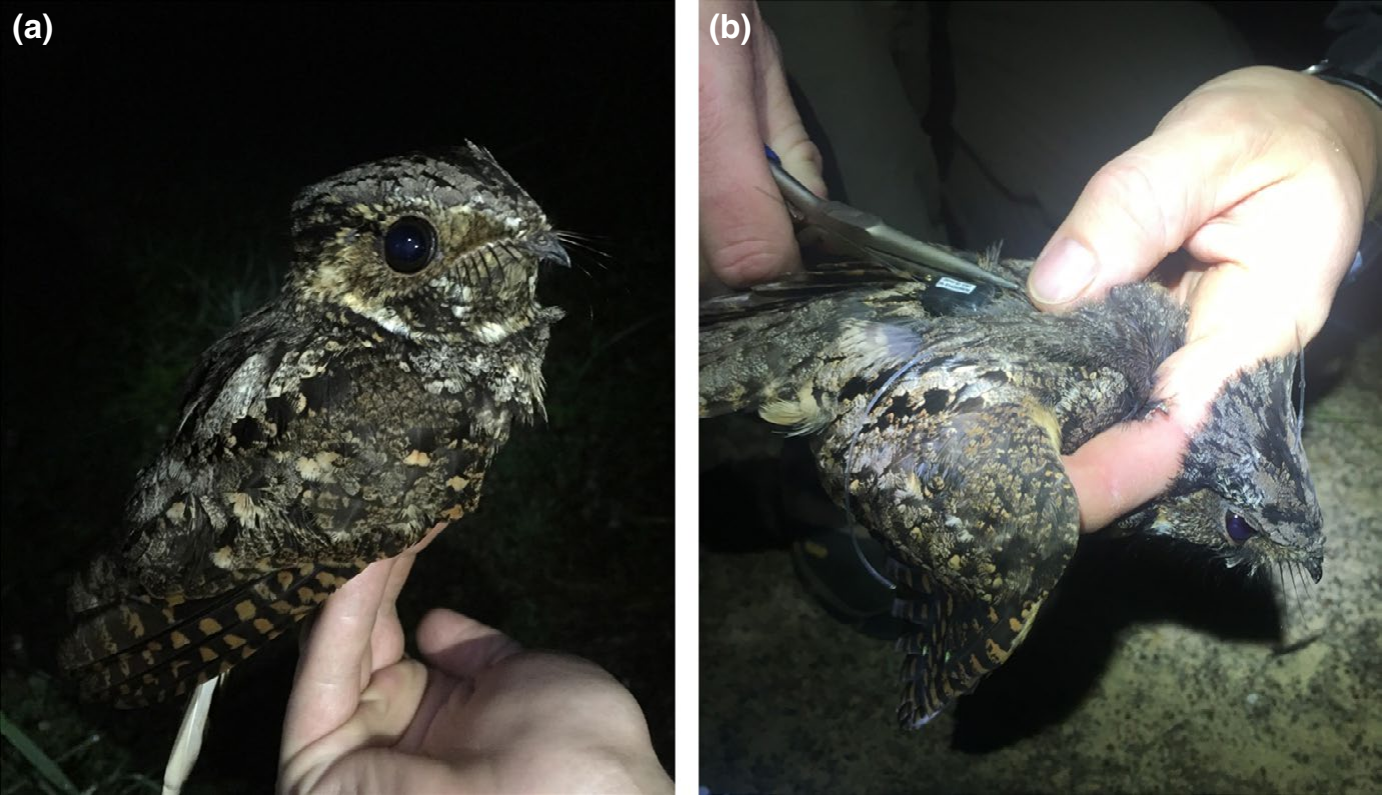
(a) An eastern whip‐poor‐will captured at Oak Openings Metropark, Toledo, OH, USA, in 2017. (b) The whip‐poor‐will is fit with a PinPoint archival GPS tag (Lotek Wireless, Inc.)

Here, we demonstrate the utility of miniaturized GPS tags in filling critical knowledge gaps in the ecology of migratory nocturnal animals. We used archival GPS tags to track a nightjar species, eastern whip‐poor‐will (Figure 1), from their breeding to their nonbreeding locations. We used the acquired spatial data from the overwintering (i.e., stationary nonbreeding) period and land cover digitized from satellite imagery to (a) determine whether or not they maintained bounded home ranges (i.e., range residency; Calabrese et al., 2016), (b) estimate home range area, (c) quantify the land cover present within their home ranges, (d) quantify land cover in the surrounding landscape (i.e., home range site), and (e) examine predictors of home range area. These results, while descriptive, are some of the first data on individual space use of a migratory nightjar during the nonbreeding period (also see Ng et al., 2018), and produce several important lines of future inquiry.

## 2 METHODS

### 2.1 Study species

The eastern whip‐poor‐will (hereafter: whip‐poor‐will; Figure 1), is a migratory member of Family Caprimulgidae that breeds in eastern North America and overwinters in the Gulf of Mexico/Caribbean Lowlands of the southern United States, Mexico, and Central America. Whip‐poor‐wills are a U.S. Fish and Wildlife Service Bird of Conservation Concern (USFWS, 2008) that have experienced sustained, enigmatic declines over most of their range (2.76% annual rate decline from 1966 to 2015; Cink, Pyle, & Patten, 2017; Sauer et al., 2017). Among the greatest areas of research need for this species is describing habitat use on the wintering grounds (Cink et al., 2017; Potter, Parnell, & Teulings, 1980). Yet, to date no published studies have used long‐distance tracking or field studies in the majority of the wintering range (i.e., outside the southeastern United States; Cink et al., 2017) to address these deficiencies. English et al. (2017) deployed light‐level geolocators to describe the nonbreeding distribution of whip‐poor‐wills from breeding sites in Canada and Korpach, Mills, Heidenreich, Davy, and Fraser (2019) used archival GPS to describe migratory routes and nonbreeding locations. However, these studies were not able to describe stationary nonbreeding space/habitat use in detail.

### 2.2 Study sites

We captured whip‐poor‐wills at two separate breeding sites in Ohio: Oak Openings Preserve Metropark (41.544°N, 83.839°W), located in northwest Ohio outside of Toledo, and Vinton Furnace State Experimental Forest (39.199°N, 82.396°W), located in southeast Ohio in the Appalachian Mountain foothills. Oak Openings Preserve is primarily an oak savanna ecosystem in the low‐lying glaciated lake plains. Vinton Furnace is a mosaic of oak‐hickory and mixed mesophytic forests in the unglaciated Allegheny plateau with narrow ridge‐and‐valley topography. Both sites are occasionally managed with controlled burns and selective logging or clear‐cuts, and consist of mature forest interspersed with open patches of shrubland or early successional forest.

### 2.3 Tag calibration, deployment, and recovery

In order to determine the accuracy of horizontal dilution of precision (HDOP) values reported for each location in each tag (model PinPoint‐10; Lotek Wireless, Newmarket, Ontario, Canada), we deployed them in a fixed location in Columbus, OH, USA (40.012009°, −83.015507°), and allowed them to collect 16 GPS points over 5 days. We then used these data for GPS error analysis when estimating home ranges (see Section 2.5 below).

Throughout the month of June 2017, we captured adult whip‐poor‐wills at the two sites in mist nets using conspecific playback between sunset and sunrise. All captured individuals received a U.S. Geological Survey aluminum band and a 1.5‐gram archival GPS tag that was between 2.5% and 3% of the bird's mass. We affixed the tags using a leg‐loop harness (Rappole & Tipton, 1991) with 0.7‐mm stretch bead cord and 1.3‐mm metal crimp beads (Figure 1). We scheduled the tags to take GPS points once every 4 days from 15 August to 15 November and every 5 days throughout the rest of the nonbreeding period. To increase the likelihood that the GPS tags would quickly locate satellites, we programmed the tags to take GPS positions at 2300 EST when birds were more likely (compared to daylight hours) to be actively foraging and thus have a clear view to the sky. We deployed tags on 6 males and 1 female at Oak Openings, and 13 males and 1 female at Vinton Furnace.

Since the GPS tags did not transmit data, it was necessary to retrieve the tags the following breeding season to access the data. We returned to the deployment locations in May and June 2018 and attempted to recapture tagged individuals using 1–3 mist nets and recordings of conspecific vocalizations. When tagged birds were not immediately captured at their previous year's territory, we expanded the capture effort to all surrounding territories where whip‐poor‐wills responded to playback. We did not mark individuals that did not receive GPS tags; thus, we only report return rates of tagged individuals.

### 2.4 Defining the overwintering period

We examined all of the location points acquired by the tags visually in Google Earth (2019) to eliminate any points that were acquired during breeding (i.e., deployment location), and movement phases (single points at locations along the spring and autumn migration routes). We then examined clusters of points acquired on consecutive days in the nonbreeding range to determine the date range of stationary nonbreeding (hereafter: overwintering). We interpreted clusters of consecutive points in a limited geographic area, following the cessation or preceding the onset of latitudinal movements (i.e., fall and spring migration), as indicative of a bird in the overwintering stage. We included all points at this location in our analysis of home range until the bird either left on spring migration (consecutive single points moving large distances northward) or the tag failed due to battery power loss (Table 1). Additionally, while most birds remained in a single geographic area, some birds relocated to a second stationary location prior to spring migration where they spent a protracted period (see Section 3 for details). However, due to the small number of points acquired at these second locations, we were only able to examine the first home range occupied for home range analysis.

**Table 1:** Summary of data and home range size analysis from archival GPS tags placed on Eastern Whip‐poor‐wills in Ohio, USA. For each bird, we report: the state and country where their overwintering home range was located, the number of points included in home range estimation, the date range of points used, and the reason for truncating location data. From the continuous time movement model (ctmm) procedures, we report the estimate of user equivalent range error (UERE) from tag calibration, the point at which the variogram asymptoted, and the movement model selected (independent identically distributed (IID), Brownian motion (BM), Ornstein–Uhlenbeck (OU), or integrated OU (IOU) movement processes). From these modeling procedures, we estimated the home range size (and lower and upper 95% confidence intervals (C.I.) in hectares based on the maximum likelihood 95% utility distribution from autocorrelated kernel density estimation; AKDE)

Bird ID	Country	State	*n*	Overwintering locations date range	Reason for date cutoff	UERE estimate (m)	Variogram asymptote	Movement model	Home range area (ha)	Lower C.I.	Upper C.I.
1757	Mexico	Oaxaca	32	17 October–21 May	Spring migration	10.93	8	IID	7.92	5.37	10.94
1758	Mexico	Chiapas	23	10 October–1 February	Relocation	8.75	7	IID	0.56	0.35	0.82
1759	Costa Rica	Puntarenas	15	8 November–27 January	Tag failure	10.34	7	IID	4.48	2.45	7.11
1760	Mexico	Tabasco	26	18 October–21 February	Tag failure	12.16	9	IID	9.46	6.13	13.52
1761	El Salvador	Usulutan	31	17 October–21 May	Spring migration	10.49	11	OUI	4.52	2.94	6.44
1764	Mexico	Chiapas	29	18 October–25 February	Tag failure	10.31	12	OUA	10.85	6.82	15.80
1768	El Salvador	Morazan	15	8 November–1 February	Relocation	10.31	7	IID	7.62	4.16	12.09
1769	Mexico	Oaxaca	27	18 October–17 February	Relocation	8.48	6	IID	0.50	0.33	0.71
1778	United States	Texas	39	2 October–21 May	Spring migration	8.68	6	IID	6.95	4.92	9.33
1779	Guatemala	Zacapa	32	14 October–9 May	Spring migration	9.20	8	OUI	1.66	1.10	2.33
1781	Mexico	Veracruz	27	26 October–25 May	Spring migration	14.69	9	IID	3.09	1.96	4.47

### 2.5 Home range estimation

We determined whether or not whip‐poor‐wills exhibit bounded overwintering home ranges (i.e., “range residents”; Calabrese et al., 2016), and the characteristics of these home ranges, using continuous time movement models (ctmm package: Fleming & Calabrese, 2015) in R (v. 3.5.2; R Core Development Team, 2018). Because of their ability to incorporate location error, movement, and serially correlated locations into the estimated utilization distributions, continuous time movement models (hereafter: ctmm) are particularly well suited to dealing with archival GPS data. We followed the workflow recommended by Calabrese et al. (2016). In brief, we first used calibration data to estimate the user equivalent range error (UERE) for each tag, such that the package could incorporate these into the error in home range estimates (Table 1). We then used the “outlie” function to identify outliers in both distance (m) and speed (m/s). We removed any extreme outliers that were likely due to tag error (0–3 points per tag). All of these points were consecutive distance (i.e., distance between two consecutive points) outliers >3 *SD* from the tag mean. In order to determine whether individuals exhibited a bounded home range, we compared variograms at different time lags and used the “ctmm.guess” function to initially estimate the variograms shape. We then compared models that either assumed no autocorrelation between points (independent identically distributed, IID), random movement (Brownian motion, BM), and either Ornstein–Uhlenbeck (OU) or integrated OU (IOU) movement processes, which assume autocorrelation among consecutive locations (summarized in Calabrese et al., 2016). We did not specify a bounded bandwidth matrix in any models. We used Akaike's information criterion corrected for small sample size (AIC_c_) to compare models. We considered the existence of an asymptote in the variogram as evidence of a bounded home range (Table 1). Once we confirmed a bounded home range, we estimated home ranges based on the best fitting model (IID, BM, OU, or IOU) using autocorrelated kernel density estimation (ADKE; Calabrese et al., 2016). Given the small sample size of points, we only estimated 95% utilization distributions for each individual.

### 2.6 Land cover data

To assess land cover composition for each bird, our workflow aimed to capture conditions close to 2017/18 when the birds were on the overwintering grounds. We digitized land cover data by hand (ArcGIS, v.10.2; ESRI, 2013) from aerial photographs, using the ESRI World Imagery (ESRI, 2019) digital globe base layer at 0.5‐m resolution as the primary, and Google Earth (2019) imagery as a secondary, source. In doing so, we built a profile of each home range that consisted of multiple aerial photographs acquired over time. Each home range profile was comprised of at least three images, with one occurring during 2017 and at least one more image after 2005. This view provided useful details of land cover change, given that we completed all digitizing via manual interpretation, since field validation was outside of the scope of this study. Importantly, the use of multiple images provides temporal window of vegetation change (Vogels, Jong, Sterk, & Addink, 2019), allowing us to identify if forest cover was removed and then subsequently regenerating, producing young forest conditions. We used the upper 95% confidence interval of the home range boundary for land cover quantification within the home range. In addition, for each bird we established a 78.54‐ha site centered on each bird's home range (i.e., a 500 m radius from the home range centroid). This scale provided a consistent means for us to make comparisons of the landscape context of each home range across individuals. The 500‐m radius circle consisted of an area at least 4× larger than any individual home range. Hereafter, we refer to this 500‐m radius area as the “site” of each home range. With limited information on the habitat and home range dynamics of eastern whip‐poor‐wills, we only evaluated these two scales (site and home range) at this time but future analysis with larger datasets should establish a multiscale perspective to address habitat selection behavior. In order to capture both the habitat within the species home range, as well as the site, we classified land cover into four classes: forest, open/cleared, scrub/shrub, and developed. Forest contained areas dominated by forest canopy. Scrub/shrub contained areas with woody vegetation that were not dominated by forest canopy (i.e., shrublands) or young forest, as determined by observing forest clearing and subsequent regeneration in the timeline of aerial imagery (see above). Open/cleared contained croplands, barren areas, pasture, and other areas dominated by nonwoody vegetation. Developed consisted of anthropogenic structures or pavement. We measured the total edge as the length (m) of all land cover class margins (McGarigal & Marks, 1995; Wang, Blanchet, & Koper, 2014) within the site (500 m radius). Total edge provides a basic measure of habitat configuration and considers edges within the site boundary (excluding the perimeter edge). In our analyses, we were not able to distinguish between soft and hard edges, but with field validation data this would be an important variable to pursue. Because field validation was not possible, we only used these general cover classes and the total edge in our formal analysis. This effort reduced error and focuses on key environmental variables that we could readily identify from the 0.5‐m resolution aerial photographs and relate to a priori factors that may influence habitat decisions at this scale.

### 2.7 Statistical analysis

We examined relationships between home range area and both intrinsic and extrinsic factors using linear models in R. As this is largely an exploratory analysis of preliminary data, we only examined simple univariate and bivariate linear models of home range area. We included the following predictors: % forest in the home range, % forest in the site, % open/clear in the site, total edge in the site (m), altitude (m), and latitude (dd). We included the last two factors to examine any geographic patterns in home range area. We limited analysis to land cover types that appeared in most/all home ranges or sites. In order to examine potential individual characteristics explaining home range area, we also included wing length as a measure of body size. Due to small sample size, we did not examine more complex models. We report all means ± standard error (*SE*).

## 3 RESULTS

**Figure 2:**
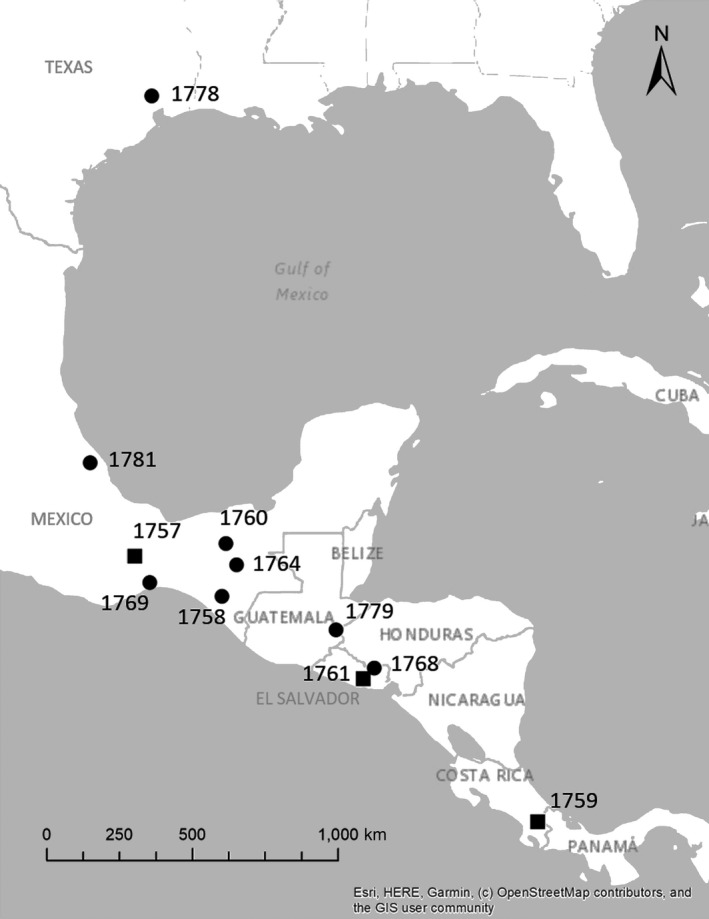
Map of the overwintering locations for eastern whip‐poor‐wills in the southern USA, Mexico, and Central America based on data from archival GPS tags deployed in Ohio, USA. in 2017 and retrieved in 2018. Squares represent locations of birds tagged in Oak Openings Metropark Reserve (northwest Ohio), and circles represent locations of birds tagged in Vinton Furnace State Forest (southeast Ohio). Numbers indicate the ID of each bird

Of the 21 archival GPS tags deployed on whip‐poor‐wills, we retrieved 11 tags (10 from males, 1 from a female), all of which successfully collected location data. Across all birds, the overwintering period occurred between 2 October and 25 March. See Table 1 for a summary of location data for each individual. Overwintering locations ranged from Texas, USA, to Puntarenas, Costa Rica, with most located in southern Mexico (Figure 2; Table 1).

**Figure 3:**
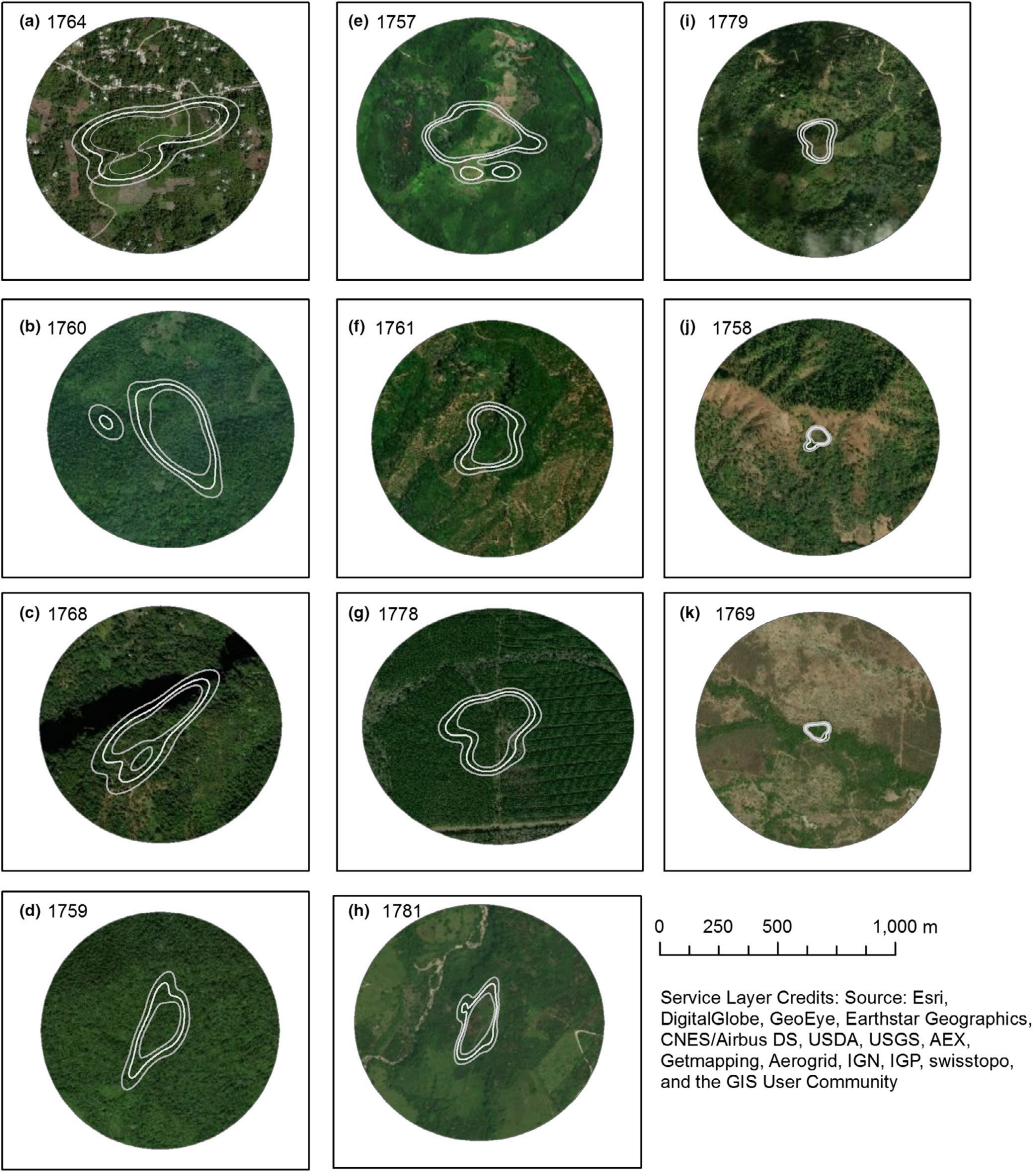
Estimates of overwintering home ranges' shape and relative size (95% autocorrelated kernel density estimates) within each home range site (500 m radius) for 11 eastern whip‐poor‐wills tracked from Ohio, USA, in 2017–2018. White lines indicate home range boundary with 95% confidence intervals, and background is aerial imagery from ESRI World Imagery (ESRI, 2019) used in digitizing land cover. The dark area in panel c is shading due to elevational differences in the landscape

**Figure 4:**
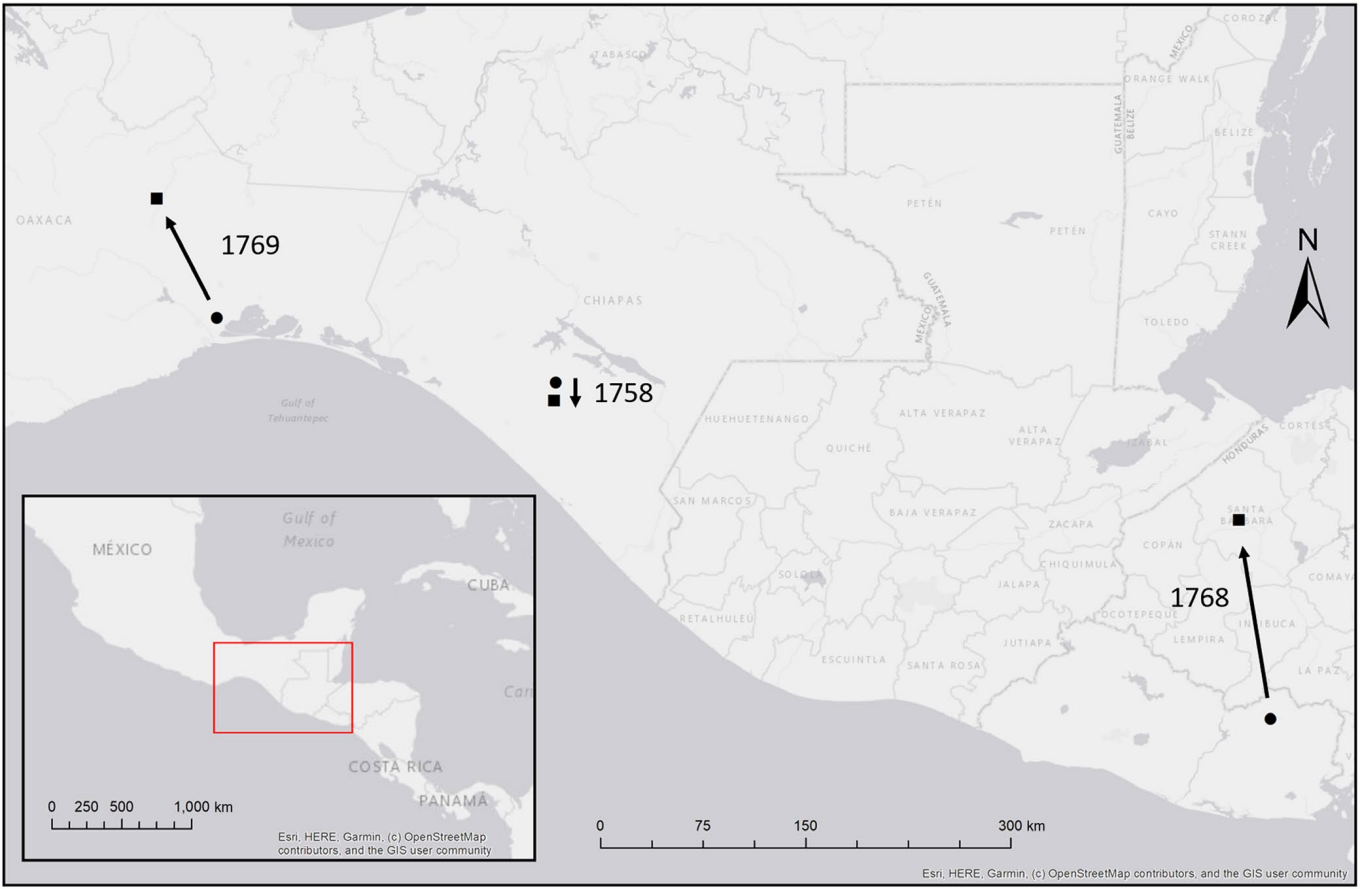
Map depicting the locations of first (circles) and second (squares) overwintering sites for three whip‐poor‐will individuals that relocated during the overwintering period based on data from archival GPS tags deployed in Ohio, USA, in 2017 and retrieved in 2018. Arrows depict the direction of movement, and numbers are individual bird IDs

All individuals exhibited at least one bounded home range during the stationary nonbreeding period (Figure 3). On average, the variograms exhibited an asymptote at 8.1 ± 0.6 locations (Table 1). There was evidence of autocorrelation in the location data of three individuals, for whom we identified the best models in ctmm as OU. For all other individuals, the model selection process did not find evidence of autocorrelation between consecutive locations; thus, we used the IID model for home range estimation (Calabrese et al., 2016; Table 1). Three individuals moved to second overwintering sites for 24, 28, and 40 days, relocating between 1.5 and 115 km (Table 1, Figure 4). All relocations occurred in early February (Table 1). We interpret this pattern as individuals using multiple overwintering sites.

### 3.1 Land cover

**Figure 5:**
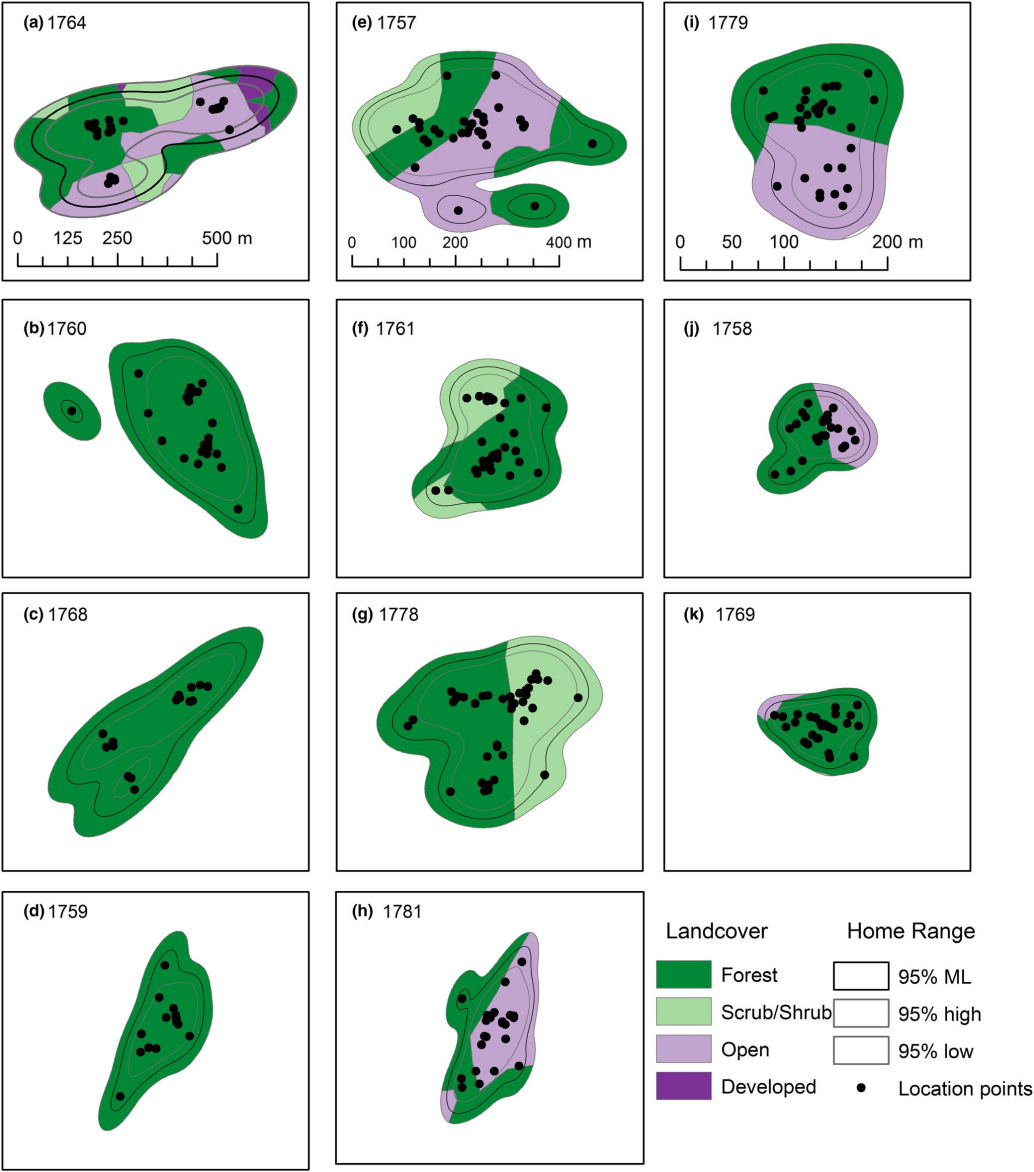
Clipped land cover classes within overwintering home ranges (95% autocorrelated kernel density estimates) of 11 eastern whip‐poor‐wills tracked from Ohio, USA, in 2017–2018. Legend defines each color‐coded land cover polygon, derived from aerial imagery acquired from ESRI World Imagery (ESRI, 2019) and Google Earth™ (2019). Each column is scaled differently (see scale bar in top row) in order to maximize visibility of variation in relative coverage of each land cover type. Points are individual locations included in the home range analysis

**Figure 6:**
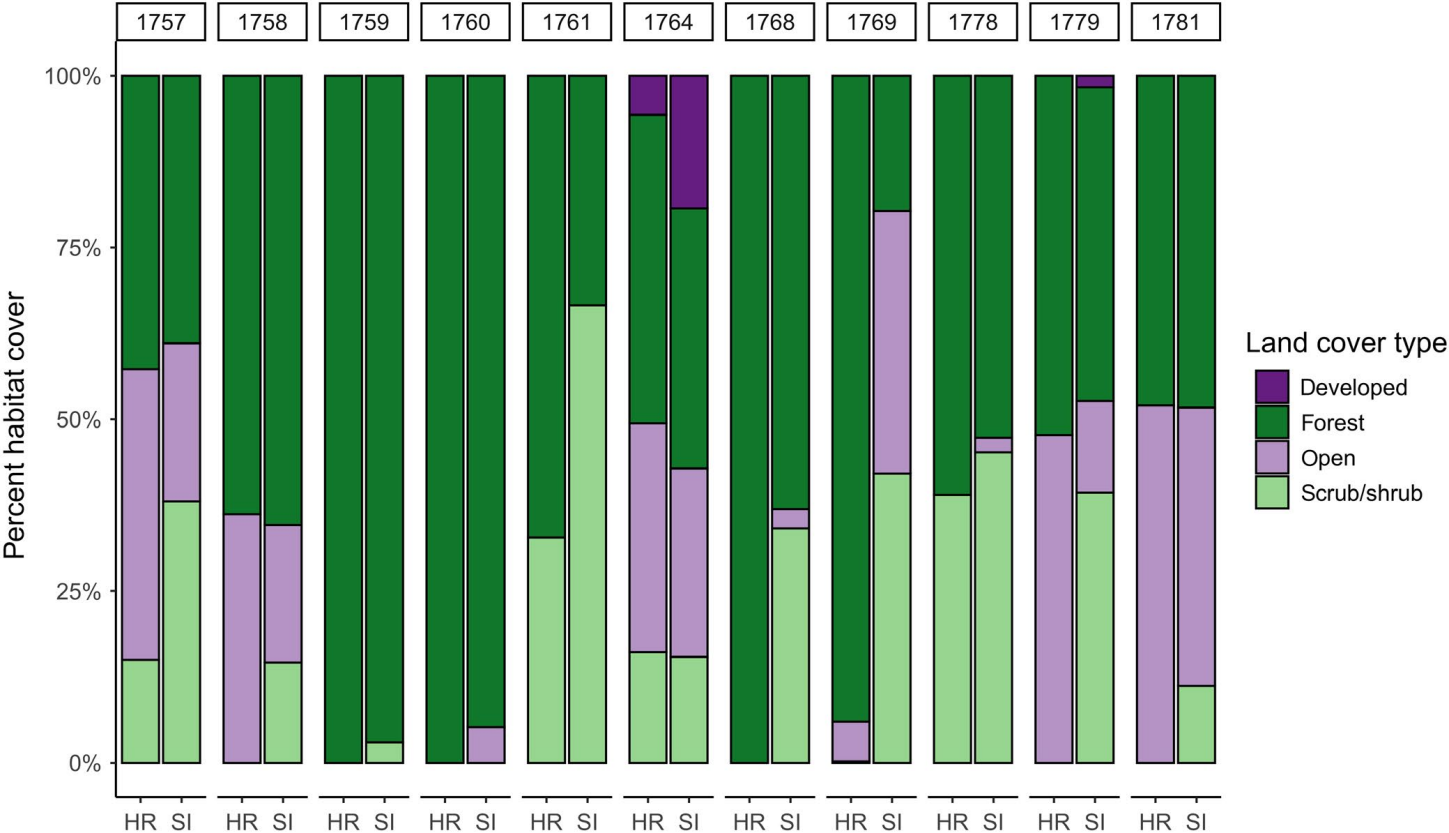
Stacked bar graphs showing proportions of each land cover type in the overwintering home ranges (bars labeled HR) and in the site of the home range (500 m radius; bars labeled SI) of 11 eastern whip‐poor‐wills tracked from Ohio, USA, in 2017–2018. See the legend for corresponding colors of each land cover type. Numbers on the *x*‐axis are individual bird IDs

Land cover within the estimated home range was variable among individuals. All individuals had some amount of forest within their home range (43%–100%; Figures 5 and 6). Nine of the 11 home ranges contained no more than two land cover types, and only one bird (with the largest home range) contained all four types (Figures 5 and 6). In all cases, each nonforest land cover constituted <50% of the home range (Figure 6). In many cases, land cover at the site scale differed from that within the home range (Figure 6). Seven individuals had more land cover types in the site than within the home range, and in nine cases, there was a higher proportion of forest cover in the home range than in the site, to variable degrees (median difference = 0.07, range = 0.03–0.74; Figure 6).

### 3.2 Home range area

**Figure 7:**
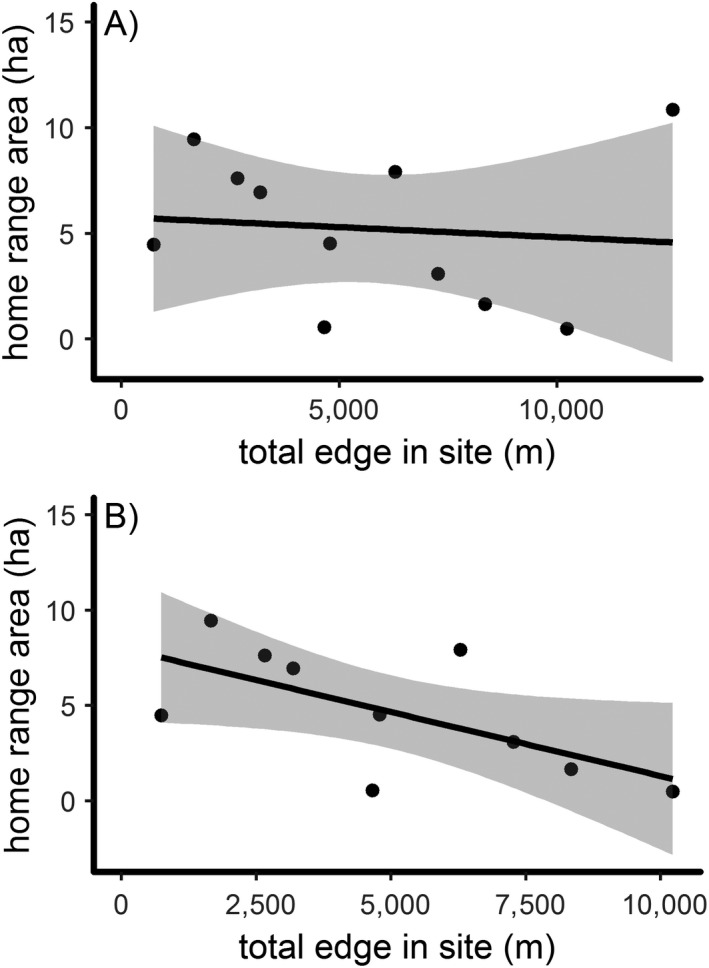
Linear relationships between overwintering home range area (ha) and total edge (m) in the site (m) (a) with the full data set (*n* = 11), and (b) excluding one outlier (*n* = 10), for eastern whip‐poor‐wills tracked from Ohio, USA, in 2017–2018

Home range area estimates ranged from as small as 0.50 ha to as large as 10.85 ha (mean: 5.24 ± 0.54 ha; Table 1, Figure 3). Our initial, exploratory analysis did not find informative predictors of home range area (all *p* > .29). However, further inspection revealed that one outlier (bird ID 1764) might be masking a relationship between home range area and total edge in the site (Figure 7a). On visual examination of the location data, this individual had multiple clusters of points with relatively large unused areas in‐between (Figure 5), possibly inflating our estimate of home range area. Thus, we re‐ran the model excluding this individual and found a negative effect of total edge in the site on home range area (*β *= −0.65, *t* = −2.32, *p* = .05; Figure 7b).

## 4 DISCUSSION

One of the greatest challenges to implementing a full‐annual cycle focus in animal ecology and conservation is revealing the basic ecology of species when they are most cryptic (Marra, Cohen, et al., 2015). Using miniaturized archival GPS tags, we were able to reveal key aspects of winter ecology in a nocturnal aerial insectivore in steep decline. Whip‐poor‐wills exhibited bounded home ranges during the overwintering period, and home ranges varied substantially in size. Most individuals' home ranges were dominated by forest canopy, and there was some limited evidence that home ranges were smaller when located in sites with more edge. While these data on only 11 individuals are largely descriptive, they reveal previously unknown aspects of whip‐poor‐will ecology outside of the breeding season. Prior to our study, nothing was known about the spatial ecology of this species at the scale of the individual home range when overwintering in the Neotropics (Cink et al., 2017; but see English et al., 2017), which constitutes the majority of their nonbreeding range.

The strong association of whip‐poor‐will home ranges with forest cover overall is not surprising, as this matches their apparent preferences during the breeding season (summarized in Cink et al., 2017). However, during breeding they can be associated with forests lacking dense understory (e.g. Wilson, 1985), and within and near clear‐cut patches of forest (Palmer‐Ball, 1996; Tozer et al., 2014). In Massachusetts, USA, Akresh and King (2016) found that whip‐poor‐wills in the breeding season were more abundant in heavily managed open‐canopy early successional forest with sparse trees, compared to closed‐canopy forest. Further, Wilson and Watts (2008) found that whip‐poor‐wills were most abundant in the breeding season in managed forest edges, compared to more mature second growth stands. These authors attributed this pattern to forest openings constituting preferred foraging habitat. Other studies suggest both young forest and closed‐canopy forest are needed to meet multiple needs (i.e., foraging and roosting; Spiller, 2019), which could explain why most individuals had at least some open or scrub/shrubland cover within their home range. Yet, given what is known about breeding habitat associations, it is surprising scrub/shrubland cover was only found in the home ranges of four individuals and never accounted for >40% of the land cover. Although many species of migratory birds are expected to use overwintering habitats that are structurally similar to their breeding sites, such assumptions have also been found to be inaccurate in some cases (e.g., Louisiana waterthrush *Parkesia motacilla*, Hallworth et al. 2011; golden‐winged warbler *Vermivora chrysoptera*, Confer, Hartman, & Roth, 2011). Similarly, in a recent study of another declining nightjar, common nighthawk, Ng et al. (2018) found that overwintering home ranges (based on roosting locations) were not associated with bodies of water, in contrast to the breeding grounds.

Although caution should be taken in interpreting our results from only 11 individuals, a number of mechanisms could potentially explain the apparent variation in habitat associations between seasons in whip‐poor‐wills. Multiple studies have demonstrated that winter space‐use patterns in Nearctic‐Neotropical migratory birds are driven by food availability (e.g., Cooper, Sherry, & Marra, 2015; Smith, Reitsma, & Marra, 2011). We expect that birds use the structural aspects of habitat as a cue for food resources (structural‐cues hypothesis; Smith & Shugart, 1987). Whip‐poor‐wills during breeding appear to time their breeding phenology with flying insect abundance, which may also limit nest survival (English, Nocera, & Green, 2018); thus, exploring relationships between food resources and overwintering land cover are warranted.

We further expect home range area to be inversely related to food availability, such that less space is needed when food is more abundant (McLoughlin & Ferguson, 2000). In our study, when excluding one outlier, whip‐poor‐will overwintering home ranges were smaller when the site of the home range contained more habitat edges. As breeding whip‐poor‐wills are known to heavily use open areas to forage (Cink et al., 2017; Eastman, 1991), perhaps overwintering individuals in more contiguous forest require larger home ranges in order to locate such microhabitats (e.g., treefall gaps). While caution should be taken in interpreting this result, it interestingly contrasts with work on the breeding grounds where no difference was found in home range area between homogeneous and heterogeneous landscapes (Wilson, 2003). This author found home ranges based on 95% utility distribution to be much larger (average > 60 ha) than what we documented during overwintering; however, another study found breeding home range sizes similar to our study (mean = 5 ha, range 1–13 ha; Hunt, 2013). During the breeding season, the primary foods of whip‐poor‐wills are beetles (Order Coleoptera) and moths (Order Lepidoptera; Garlapow, 2007), but their diet in winter is not well described. More research is needed to determine whether the apparent seasonal difference in habitat associations and home range size is due to differences in food resources and/or energetic requirements, or whether the habitat of their preferred food is different. One important caveat to our findings, however, is that while we assume the timing of our location points coincides with foraging activity, some locations may be nocturnal roost/resting locations. This may be especially of note on nights with little to no moonlight, which we did not account for, as this appears to drive foraging activity on the breeding grounds (English et al., 2018).

In addition to food availability, seasonal variation in intra‐ or interspecific competition could also drive variation in space use between seasons. For example, adult male American redstarts (*Setophaga ruticilla*) displace females and subadult males from higher quality into lower quality habitats when they arrive on the overwintering grounds (Marra, 2000). Thus, the social context of an individual whip‐poor‐will's home range may be a stronger factor in the selection of habitats than the preferences of the individual. In addition, multiple resident nightjar species may compete with whip‐poor‐wills for resources within the overwintering region we documented, including buff‐collared nightjar (*Antrostomus ridgwayi*) and common pauraque (*Nyctidromus albicollis*). Both of these species are found breeding in second growth and scrub habitats, which are similar structurally to the apparently preferred breeding habitat of whip‐poor‐wills (Howell & Webb, 1995). As others have noted (e.g., Greenberg, 1995; Johnson, Sherry, Strong, & Medori, 2005), competition with resident species can drive overwintering habitat use in migrants through competitive exclusion. While our remote tracking of individuals revealed much about the overwintering context of whip‐poor‐wills, without in situ studies of populations of interacting individuals, this level of understanding is not possible.

Interestingly, three of the birds we tracked appeared to occupy multiple overwintering sites. There is the possibility additional birds in our sample exhibited similar behavior, but tag failure precluded us from detecting it. This adds to a growing body of literature documenting such within‐season relocations during the overwintering period in migratory birds (e.g., Renfrew et al., 2013; Stutchbury et al., 2016), including recently in another nightjar species (common nighthawk; Ng et al., 2018). Additional tracking data are needed to determine the prevalence of such movements in whip‐poor‐wills, and any individual or environmental factors driving it. It is notable that all of the relocations we observed occurred at similar times (early February), and that the individuals with the two smallest home ranges both relocated. Stutchbury et al. (2016) tested competing hypotheses for nonbreeding intraseasonal movements in another aerial insectivore (purple martin *Progne subis*). These researchers found support for the “competition‐avoidance hypothesis,” but not the “resource hypothesis,” suggesting martins were responding to changes in competitor density, as opposed to resource declines. Given these relocations occur as the breeding season for resident species approaches, this provides another incentive to explore interspecific competition in this system.

Studies such as this one are critical for better understanding population limitation across the full‐annual cycle. Integrated population models (IPMs; Hostetler, Sillett, & Marra, 2015; Rushing et al., 2017) incorporate demographic and vital rate data from different seasons to determine their relative influence on population growth. Thus, it is now possible to quantitatively isolate where and when populations of migratory birds are most limited, but these models require information about within‐season dynamics. An important next step is to quantify migratory connectivity (the degree to which populations remain discrete between breeding and nonbreeding seasons; Webster et al., 2002) for this species. Based on our results, the population spread in winter appears to be high (Finch, Butler, Franco, & Cresswell, 2017), suggesting low connectivity; however, tracking data from more sites are required for a quantitative estimate of migratory connectivity (Cohen et al., 2018; but see English et al., 2017; Korpach et al., 2019). This is critical data for IPMs, as data must be collected on linked breeding and nonbreeding populations, in order to generate relevant estimates of seasonal vital rates. This study and others (e.g., English et al., 2017; Korpach et al., 2019) have demonstrated the utility of archival tags for whip‐poor‐wills. The use of archival GPS in particular will maximize the precision of location data and allow more broadscale studies of space use outside the breeding season to inform demographic models. Difficult to detect species present an enormous challenge for conservation and animal ecology, particularly across the full‐annual cycle. However, the “golden age” of animal tracking which we are currently experiencing (Kays, Crofoot, Jetz, & Wikelski, 2015) has given researchers the ability to overcome such challenges for many species.

## CONFLICT OF INTEREST

We have no competing interests.

## AUTHOR CONTRIBUTIONS

Tonra wrote the first draft of the manuscript, contributed to study design, conducted home range analysis, and edited the manuscript. Wright conducted all fieldwork, contributed to study design and analysis, and helped edit the manuscript. Matthews acquired funding, conducted land cover analysis, contributed to study design, and helped edit the manuscript.

## ETHICAL APPROVAL

All fieldwork involving animals was approved by the Institutional Animal Care and Use Committee of The Ohio State University (protocol 201500000028), and was conducted under a Master Banding Permit issued by the U.S. Geological Survey Bird Banding Laboratory to Tonra (#23941).

## Data Availability

All locations are available upon request and will be publically available on http://www.Movebank.org beginning January 2021 (https://doi.org/10.5441/001/1.6v110sv8; Tonra, Wright, & Matthews, 2021).
